# Elimination of saphenous reflux after tributary sclerotherapy: report of two cases

**DOI:** 10.1590/1677-5449.200064

**Published:** 2021-06-11

**Authors:** Felipe Puricelli Faccini, Claudia Carvalho Sathler-Melo

**Affiliations:** 1 Hospital Moinhos de Vento – HMV, Cirurgia Vascular, Porto Alegre, RS, Brasil.; 2 Instituto de Cardiologia, Porto Alegre, RS, Brasil.; 3 Angiovasc, Belo Horizonte, MG, Brasil.

**Keywords:** saphenous vein, sclerotherapy, venous reflux, venous insufficiency, safena, escleroterapia, refluxo venoso, insuficiência venosa

## Abstract

Most patients with chronic venous disease (CVD) and reflux in the saphenous vein are treated with saphenous stripping or ablation. The venous hemodynamics approach offers the possibility of treating saphenous reflux without eliminating the saphenous vein. We present 2 cases in which venous reflux was eliminated while preserving the great saphenous vein, after treatment with hemodynamic sclerotherapy using a protocol of synergic use of Dextrose and long pulse Nd YAG 1064 laser. These cases show that treating the tributaries responsible for saphenous reflux can correct hemodynamic imbalances and restore normal flow in the great saphenous vein with improvements in symptoms and esthetics. Long-term results are still uncertain.

## Resumo

A maioria dos pacientes com insuficiência venosa crônica e refluxo na veia safena é tratada com retirada ou ablação da safena. A hemodinâmica venosa traz a possibilidade de tratar esses pacientes sem eliminar a veia safena. Nós apresentamos dois casos de refluxo parcial de veia safena magna resolvidos com escleroterapia hemodinâmica. Usamos um protocolo de uso sinergístico de glicose 75% e Nd-YAG *laser* 1064. Os casos nos mostram que o tratamento das tributárias pode corrigir o refluxo da veia safena e obter melhora clínica e cosmética. Os resultados de longo prazo ainda são incertos.

## INTRODUCTION

Resolution of reflux in the deep venous system after cessation of reflux in the superficial system has been common knowledge for a long time.^1^ The same principle applies to different levels of the venous compartment, such as the saphenous vein. Adequate assessment and evaluation of venous reflux is a long-standing concern in the vascular community.^2^ Despite significant advances in duplex techniques and technology, treatments have changed little over the last decades and still consist of eliminating all involved veins. Decades ago, Franceschi showed that reflux in the saphenous vein can be resolved without eliminating the vein itself, with CHIVA.^3^ Years later, the ASVAL technique has made a further contribution, showing that the saphenous vein can be spared.^4^ We consider that the rationale of both techniques can contribute to improved sclerotherapy results.

Modern techniques have made saphenous procedures much easier than before, resulting in increased numbers of saphenous treatments and treatment of patients at lower CEAP classes. Studies report increases in the number of saphenous vein procedures, most of them performed by “non-traditional” specialists dealing with CVD.^5^^,^ 6 Baber et al. showed that physicians who do not traditionally treat chronic venous disease and high-volume providers are more likely to do endovenous therapy.^5^ A recent study showed that saphenous procedures increased by 0.83% every year from 2007 to 2017 in Belgium. The same study showed that patients with limited financial resources (preferential reimbursement) had significantly lower intervention rates than patients on the usual reimbursement system.^7^

The long-term effects of this increase in saphenous vein elimination are unclear, as clinical trials do not extend beyond 5 years.^8^ The long-term results of the novel approaches are similar to the results of the stripping.^9^^,^ 10 Studies have shown that the rate of recurrence after saphenous stripping is close to 60% after 30 years.^11^ This causes enormous patient burden and medical costs. We present 2 cases in which we eliminated saphenous reflux with hemodynamic sclerotherapy of collaterals. The total reversal of saphenous reflux makes us rethink the modern trend for saphenous destruction and its effects on recurrence and quality of life in the long run. The Research Ethics Committee approved this study (decision number 4.723.760).

## CASE REPORTS

### Case 1

**Figure 1:**
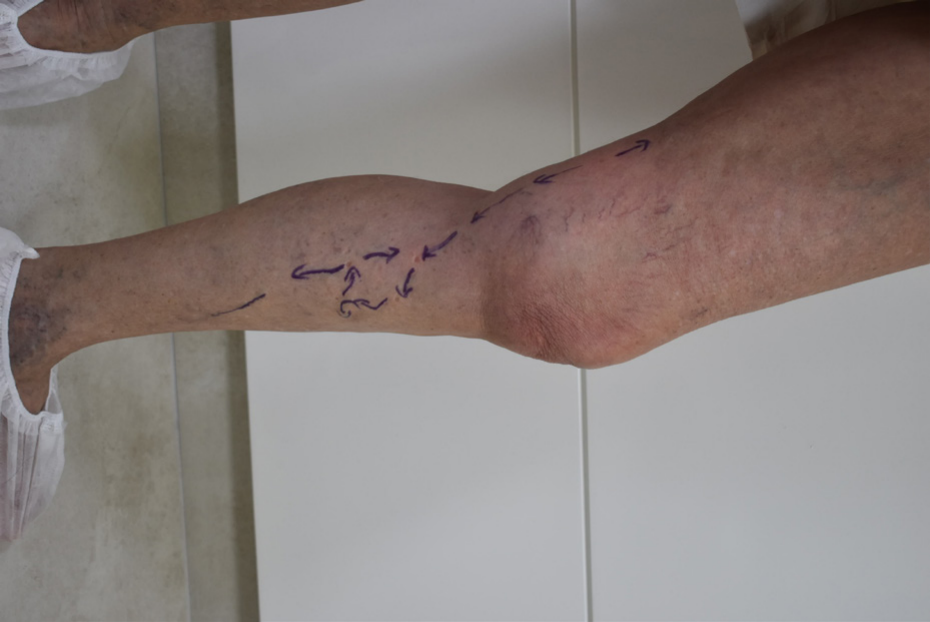
Hemodynamic schema. Reflux in the saphenous vein distal to a collateral at the thigh, transferred to a leg collateral. The same collateral then transfers reflux back to the saphenous vein a few centimeters distally.

**Figure 2:**
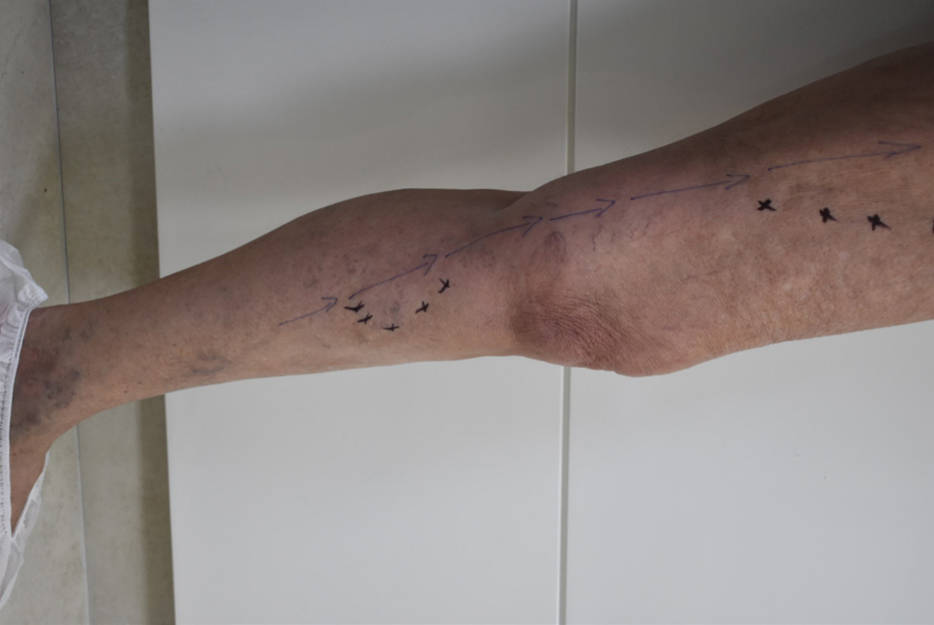
Hemodynamic schema. The saphenous vein no longer has reflux and the collaterals involved are occluded. Observe that the veins at the ankle have regressed without direct treatment.

A 64-year-old woman with chronic venous disease (CEAP class C3), and history of 2 previous venous operations on the leg. Both operations were phlebectomies and neither procedure involved treatment of the saphenous veins (our Duplex ultrasound was equal to an exam prior to the previous operation). The hemodynamic evaluation identified a 3 mm collateral at the mid-thigh transferring reflux to the 5.8 mm great saphenous vein, which presented reflux from this point to the upper leg. Distally, the saphenous vein was transferring reflux to a 3.5 mm tributary, from which point it resumed upward flow for a few centimeters, until the same collateral transferred reflux to the GSV again. We present the pre-treatment hemodynamics of this case in Figure 1 and post-treatment in Figure 2. We proposed fractionation of the collaterals according to CHIVA principles using sclerotherapy. We performed the protocol as described below. The patient returned 2 weeks and 3 months after the procedure with total improvement of symptoms and some esthetic improvement. The duplex scan showed that sclerotherapy had ablated the large collaterals. The GSV reflux had been eliminated and flow had normalized (with no reflux) after closure of the collaterals. It had also reduced in size from 0.58 mm to 0.52 mm.

### Case 2

**Figure 3:**
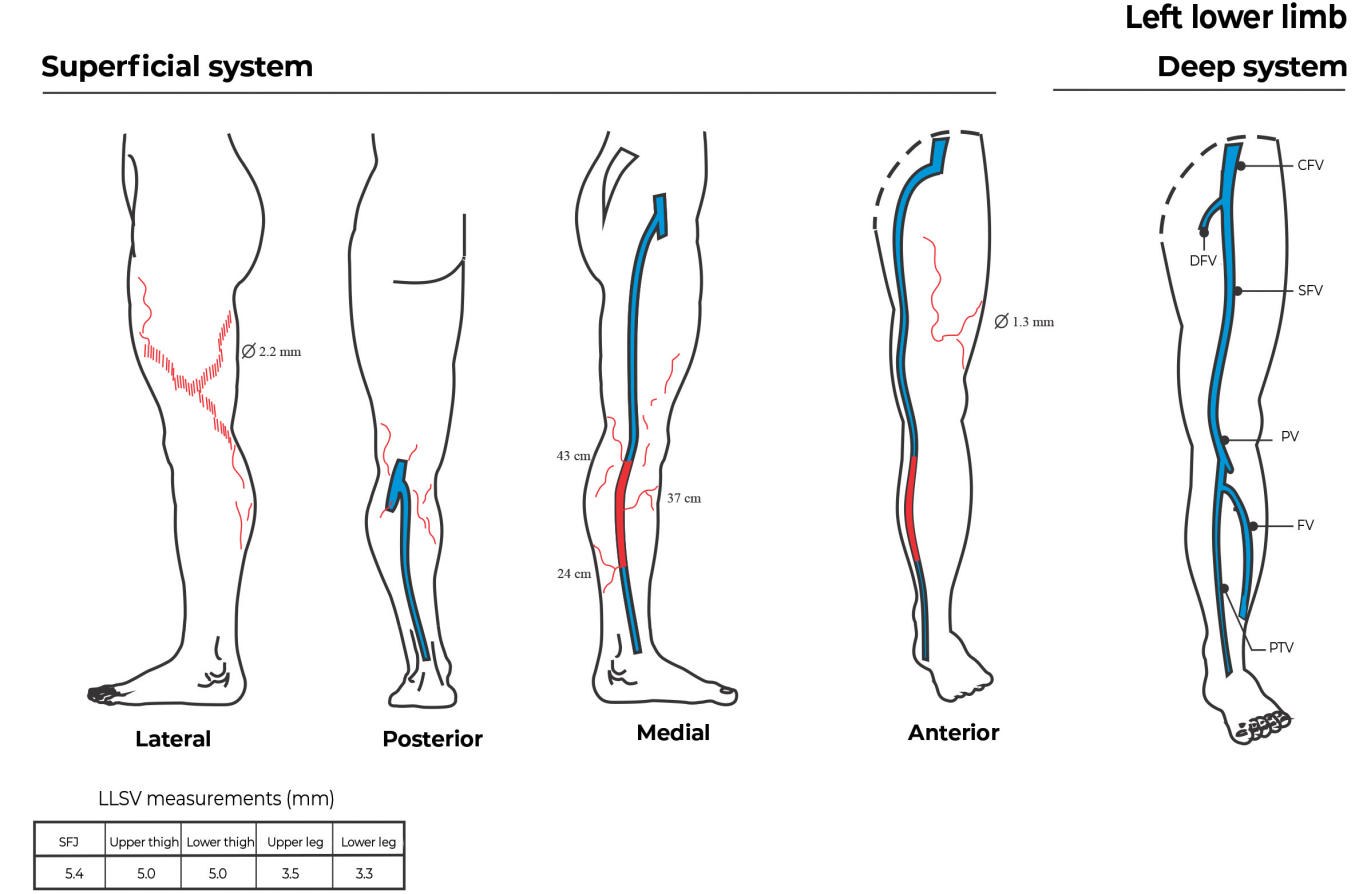
Pre-treatment - Red veins represent the portions of saphenous vein and collaterals with reflux. CFV: Common femoral vein; DFV: Deep femoral vein; SFV: Superficial femoral vein; PV: Popliteal vein; FV: Fibular vein; PTV: Posterior tibial vein; LLSV: Lower limb superficial veins; SFJ: saphenofemoral junction.

**Figure 4:**
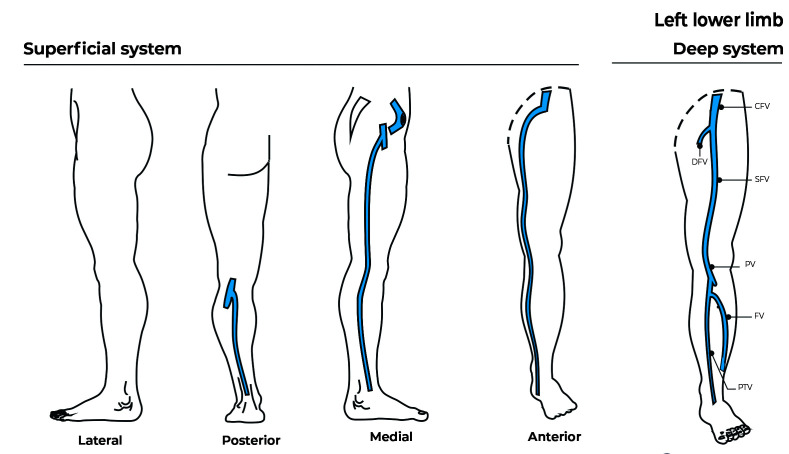
Post-treatment - The saphenous vein has upward flow and no reflux and the collaterals have disappeared. CFV: Common femoral vein; DFV: Deep femoral vein; SFV: Superficial femoral vein; PV: Popliteal vein; FV: Fibular vein; PTV: Posterior tibial vein.

A 34-year-old asymptomatic woman with chronic venous disease (CEAP class C2), presenting with cosmetic complaints only. She had no history of previous treatments. Hemodynamic evaluation of the left lower extremity showed reflux at the great saphenous vein from one tributary at the knee level to another at the mid-leg (Figure 3 and Figure 4, pre-treatment and post-treatment). She had reticular veins and telangiectasias on the lateral aspect of the thigh and calf. We proposed Hemodynamic CLaCS according to the protocol described below. We performed 2 sessions in 3 months and at 4 weeks after the second session the veins had disappeared and the patient was satisfied with the cosmetic results. The duplex scan showed absence of reflux in the great saphenous vein and occlusion of the collaterals involved in the reflux.

### Hemodynamic CLaCS protocol

We treat CEAP class C1-C3 patients by sclerotherapy of collaterals according to CHIVA principles as previously described;^12^^-^ 14 we treat higher CEAP classes or failures of sclerotherapy with saphenous-sparing operations. The authors have adapted a technique proposed by Miyake et al. to treat smaller veins.^15^ The procedure comprises using Nd-YAG laser 1064 shots at the collateral prior to injection of Dextrose 75% sclerosing agent to create a synergic effect. The laser causes the vein to contract, minimizing the volume of Dextrose administered. The contraction and the low volume prevent backflow to the saphenous vein, thus we only take measures to prevent it in cases in which a deep portion of the vein requires sclerotherapy. We use a spot size of 3-9 mm depending on the vein size. We have adapted the usual CLaCS protocol^15^ and treated veins with larger diameter with a few energy delivery changes. A 3mm spot is used for veins < 0.10 mm, a 6mm spot is used for those <2.5mm, and a 9mm spot is used for collaterals >2.5 mm. Fluency varies from 40 to 90 J/cm and the pulse range is 15-50 Msec. Local administration of cool air is used to minimize pain. After treatment of a few centimeters with the laser, we inject cold 75% dextrose into portions of the vein that remain open. Deep superficial thigh veins receive ultrasound-guided Dextrose injections and laser and dextrose in superficial parts. Both injections and laser follow the roadmap of augmented reality equipment.^16^ We call the procedure Hemodynamic CLaCS (Cryo Laser Cryo Sclerotherapy), in reference to the protocol of synergic use of laser and dextrose and the hemodynamic principles applied.

## DISCUSSION

Technological developments have made it easier to eliminate the saphenous vein than it was in the past. There has been an increase in the number of saphenous veins treated over the last few years.^5^^-^ 7 ^,^ 17 Long-term recurrence after endovenous saphenous treatment is uncertain and the 5-year results match the results of stripping procedures.^18^ Basic and clinical studies have shown that vein elimination can trigger recurrence of varicose veins.^19^^,^ 20 We therefore consider that strategies to minimize saphenous elimination while improving symptoms and the cosmetic appearance of the leg are welcome.

Widespread use of duplex ultrasound has led to diagnosis of many cases of reflux that would have gone unnoticed in the past. We should remember that reflux is not a disease per se. Engelhorn et al. found that women with telangiectasias (CEAP Class C1) had saphenous vein reflux detected in 46% of their extremities.^21^ Patients with symptomatic CEAP class C0-C3 with reflux may improve with exercises, weight loss, and quality-of-life measures. Phlebotonic drugs may also play a role in managing these patients.^22^ We should remember that reflux per se is not a mandatory indication for any procedure and, above all, not for saphenous elimination. A recent editorial reminds us that patients can choose to do nothing and should be made aware of this.^23^

When simpler treatments cannot solve the problems, another intervention may be necessary. We spare the saphenous vein in these cases using CHIVA, with good clinical hemodynamic and cosmetic results.^12^ The ASVAL technique is also used in patients with a single collateral involved in saphenous reflux.^4^ In simpler cases with low CEAP, elimination of saphenous collaterals aspirating blood or causing focal venous hypertension may prevent the venous system from deteriorating. We presented cases in which we resolved saphenous reflux by sclerotherapy of collaterals.

The prevalence of telangiectasias in the general population is high and these patients may have symptoms.^24^^,^ 25 Thus, treatment and ultrasound examination may help CEAP Class C0-C3 patients, if simpler non-interventional treatments fail. Patients with only aesthetic complaints may also benefit from simpler treatments that do not eliminate the saphenous vein. Many patients with esthetic-only complaints and CEAP class C1-C3 have reflux that is detected by a duplex scan. Our cases show that the reflux per se is not a sine qua non justification for saphenous ablation. Hemodynamic sclerotherapy may achieve good esthetic/clinical results.

Successful flow reversal in the saphenous vein depends on a few factors. The competence of the terminal femoral valve is important because it breaks the height of the blood column. In patients with important femoral reflux, flow may not reverse after treatment of the collaterals. Physical characteristics of the vein also play a role, since we have observed that the longer and larger the incompetent vein, the more difficult it is to correct the reflux, and the longer and larger the competent distal vein, the easier it is to reverse the flow. Direct saphenous perforators are also decisive to maintaining reflux. Successful reversal of flow in the great saphenous vein is impressive, but patients still experience improvement of symptoms with sclerotherapy even if reflux remains.^14^

If we consider the saphenous vein as a conduit for the flow, we should also consider that by stopping the reflux's pressure gradient, we can recover it. In this paper, we presented cases in which reflux was successfully resolved just by eliminating collaterals with Laser/Dextrose sclerotherapy. We consider that the procedure is a suitable alternative option for treating cases with low CEAP classes, but long-term results are not yet available. Using sclerotherapy with foam in the setting of hemodynamics is not new.^14^ However, the laser synergy contracts the vein and makes it possible to obliterate the vein with less sclerosing volume. We consider this approach to be a low-cost and low-risk alternative that makes it possible to preserve the saphenous vein in some patients.
